# Essential oils extracted from nine different plants exhibit differential effects on skin antioxidation and elasticity

**DOI:** 10.1002/2211-5463.13778

**Published:** 2024-02-26

**Authors:** Da Som Kim, Min Jae Kim, Mi‐Jin Park, Byoung‐Jun Ahn, Wook‐Joon Yu, Sung‐Min An, Beum‐Soo An

**Affiliations:** ^1^ Department of Biomaterials Science (BK21 FOUR Program), College of Natural Resources and Life Science/Life and Industry Convergence Research Institute Pusan National University Miryang Korea; ^2^ Developmental and Reproductive Toxicology Research Group Korea Institute of Toxicology Daejeon Korea; ^3^ Division of Forest Industrial Materials, Department of Forest Products and Industry National Institute of Forest Science Seoul Korea; ^4^ Division of Endocrinology, Department of Internal Medicine University of California Davis School of Medicine CA USA

**Keywords:** antioxidant, essential oils, *Juniperus chinensis* L., *Juniperus chinensis* var. *sargentii*, medicinal plant, skin elasticity

## Abstract

Essential oils derived from plants are major ingredients in the medical and cosmetic industry. Here, we evaluated nine types of plant essential oils to identify potential candidates with antioxidant and elasticity‐enhancing properties. Seven essential oils showed at least 10% radical scavenging activity at the highest concentration. Essential oils extracted from *Aster glehnii*, *Cinnamomum cassia*, *Citrus unshiu*, *Juniperus chinensis* L., and *Juniperus chinensis* var. *sargentii* significantly enhanced fibroblast viability, and oils from *Cit. unshiu*, *J. chinensis* L., and *J. chinensis* var. *sargentii* significantly increased cell proliferation and migration. Expression of extracellular matrix proteins, including collagen 1, collagen 3, and elastin, were upregulated by *J. chinensis* L. and *J. chinensis* var. *sargentii* oil, which also significantly enhanced the contractile activity of skin cells in a three‐dimensional gel contraction assay. The results suggest that *J. chinensis* L. and *J. chinensis* var. *sargentii* essential oils may be potential anti‐wrinkling and anti‐oxidative agents for future consideration of use in the medical and cosmetic industry.

## Graphical abstract

This study evaluated nine types of plant essential oils to select ingredients with antioxidant and elasticity‐enhancing properties. *Juniperus chinensis* L. and *J. chinensis* var. *sargentii* essential oils increased the antioxidant ability of fibroblasts and promoted their proliferation, thereby increasing production of proteins related to skin elasticity and contractile ability. These essential oils are potential anti‐oxidative agents.

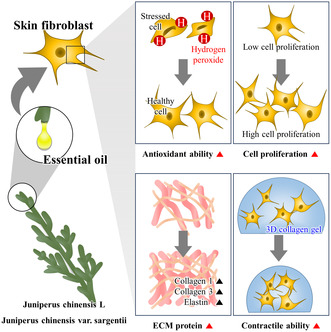

**Abbreviations**
3D: three‐dimensional
ABTS: 2,2′‐azino‐bis‐(3‐ethylbenzothiazoline‐6‐sulfonate)
BrdU: 5‐bromo‐2‐deoxyuridine
DW: distilled water
ECM: extracellular matrix
FBS: fetal bovine serum
H_2_O_2_
: hydrogen peroxide
IC_50_
: half maximal inhibitory concentrations
MEM: modified Eagles' medium
MTT: 3‐(4,5‐dimethylthiazol‐2‐yl)‐2,5‐diphenyl tetrazolium bromide
NHDFs: normal human dermal fibroblasts
OD: optical densities
ROS: reactive oxygen species
SD: standard deviation

The medical and cosmetics market for skin health products has expanded in parallel with improved living standards and increased average life expectancy [1]. Antioxidant ability is the most important function required for functional cosmetic products that improve skin health [2]. When the skin is exposed to internal factors such as aging or external factors such as ultraviolet rays, reactive oxygen species (ROS) are generated in skin, and this ROS inhibits fibroblast proliferation in the dermal layer and the ability of fibroblasts to produce extracellular matrix (ECM) [3, 4]. Fibroblasts maintain skin elasticity, an indicator of skin health, by producing ECM proteins, such as collagen and elastin. Therefore, a decline in fibroblast activity results in a failure to synthesize ECM proteins and a consequent decrease in dermal ECM protein levels [5]. Of the various types of ECM proteins, collagen 1, collagen 3, and elastin account for the largest amount of protein in the dermal layer and are the primary skin components responsible for maintaining skin elasticity [6, 7]. Therefore, it is important for skin care products and cosmetics to contain ample collagen to eliminate ROS and enhance skin elasticity.

Currently, the active anti‐oxidative and anti‐wrinkle ingredients of functional cosmetics include tocopheryl acetate, tocopherol, ascorbic acid, niacinamide, and retinyl palmitate [8]. However, these are mostly organic synthetic materials that can cause skin irritation or have stability problems, and consequently, their use is being increasingly prohibited or restricted [9, 10]. Additionally, as the demand for mild and nature‐friendly raw materials has increased, interest in natural products is rising, and cosmetic companies are actively researching to replace problematic organic synthetics with natural alternatives [11].

Natural products are mostly composed of plant, animal, and microorganism secondary metabolites and some primary metabolites [12] and are biodegradable and usually have mild continuous actions, which results in few side effects and environmental friendliness [13, 14]. Plant natural products include many substances with antioxidant activities, such as essential oils [15]. These oils have strong aromatic properties and widely varied compositions and contents, which are contingent upon the unique characteristics of the plants [16]. For example, essential oils extracted from *Pistacia atlantica* have antioxidant effects on skin cells, while those extracted from lemons, juniper, and grapefruit have anti‐wrinkle effects [16, 17]. Also, essential oils extracted from the Manuka tree and *Zingiber officinale* Roscoe have anti‐wrinkle effects in mice [18].

As mentioned above, many essential plant oils have beneficial effects on skin. In this study, we screened Korean traditional native plants that showed potential for antioxidant efficacy from literature references [19, 20, 21, 22, 23, 24, 25]. Afterwards, plants for which the optimal concentration that could exhibit functionality in skin cells without causing cytotoxicity had not been identified were selected and used in this experiment. Thus, we analyzed and compared the antioxidant and physiological effects of nine plant essential oils on skin.

## Materials and methods

### Preparation of essential oils from nine types of plant

As shown in Table 1, the essential oils of nine medicinal plants were prepared by hydrodistilling different plant parts (National Institute of Forest Science, Republic of Korea). Each sample was mixed with distilled water (DW) in a ratio of 1 : 10 (kg : L). Mixtures were then heated at 102 °C using a heating mantle (cat. no. MS‐DM608; Misung Scientific, Gyeonggi‐do, Korea), and volatiles were condensed using a Dean‐Stark trap (National Institute of Forest Science, Republic of Korea). Finally, the essential oils were dehydrated using anhydrous sodium sulfite and stored at 4 °C until required.

**Table 1:** Scientific names, abbreviations, common names, and plant parts used.

No.	Scientific name	Abbreviation	Common name	Parts used
1	*Aster glehnii* F. Schmidt	*A. glehnii*	Ezo‐goma‐na	Grass clumps
2	*Artemisia capillaris*	*Ar. capillaris*	Yin Chen Hao	Grass clumps
3	*Cinnamomum cassia*	*C. cassia*	Chinese cinnamon	Leaves
4	*Citrus natsudaidai* Hayata	*Cit. natsudaidai*	Natsumikan	Peels
5	*Citrus pseudo gulgul*	*Cit. pseudo gulgul*	Hill lemon	Peels
6	*Citrus unshiu*	*Cit. unshiu*	Satsuma orange	Peels
7	*Juniperus chinensis* L.	*J. chinensis* L.	Chinese juniper	Leaves
8	*Juniperus chinensis* var. *sargentii*	*J. chinensis* var. *sargentii*	Sargent juniper	Leaves
9	*Zanthoxylum piperitum*	*Z. piperitum*	Japanese pepper	Fruits

### Cells and cell culture

Normal human dermal fibroblasts (NHDFs) were purchased from American Type Culture Collection (Manassas, VA, USA) and cultured in modified Eagles' medium (MEM; Welgene, Daegu, Republic of Korea) containing 10% fetal bovine serum (FBS; Sigma‐Aldrich, St. Louis, MO, USA) and 1% streptomycin/penicillin in a 5% CO_2_ incubator at 37 °C until the cell confluence reaches 100%. Cells were counted, and 5 × 10^4^ cells/well were cultured in 24‐well plates for MTT and BrdU assays for 24 h, and 2 × 10^5^ cells/well were cultured in 6‐well plates for western blot for 24 h. The cells were then treated with the essential oils for 24 h.

### Determination of half maximal inhibitory concentrations (IC_50_) and highest non‐toxic concentration

An 3‐(4,5‐dimethylthiazol‐2‐yl)‐2,5‐diphenyl tetrazolium bromide (MTT) assay was performed to determine cell viabilities and IC_50_ values of the nine essential oils, as we previously described [26]. NHDFs were treated with the nine essential oils at various concentrations (Table 2) in culture medium in a 5% CO_2_ incubator for 24 h at 37 °C. After removing culture media, cells were treated with 500 μL of 0.5 mg·mL^−1^ MTT (Sigma‐Aldrich) and incubated in a 5% CO_2_ incubator for 4 h at 37 °C. MTT solution was then removed, 300 μL of DMSO was added to dissolve formazan crystals, and optical densities (OD) were measured at 570 nm using a microplate reader (Agilent Technologies, Waldbronn, Germany). Cell viability ratios were calculated using: OD sample/OD control × 100, and IC_50_ values were calculated using sigmaplot v.10.0 (Systat Software, San Jose, CA, USA).

**Table 2:** Concentrations of plant essential oils used for each experiments.

No.	Essential oil	Tested concentrations (p.p.m.)						
		MTT assay (IC_50_)	ABTS assay	MTT assay (H_2_O_2_)	BrdU assay	Scratch migration assay	Western blot analysis	Collagen gel contraction assay
1	*Aster glehnii*	6.25, 12.5, 25, 50, 100	25	25	25	25	25	25
2	*Artemisia capillaris*	1.5625, 3.125, 6.25, 12.5, 25	3.125	3.125	–	–	–	–
3	*Cinnamomum cassia*	12.5, 25, 50, 100, 200	50	50	50	50	50	50
4	*Citrus natsudaidai*	6.25, 12.5, 25, 50, 100	12.5	12.5	–	–	–	–
5	*Citrus pseudo gulgul*	6.25, 12.5, 25, 50, 100	25	25	–	–	–	–
6	*Citrus unshiu*	6.25, 12.5, 25, 50, 100	25	25	25	25	25	25
7	*Juniperus chinensis* L.	12.5, 25, 50, 100, 200	25	25	–	–	–	–
8	*Juniperus chinensis* var. *sargentii*	12.5, 25, 50, 100, 200	25	25	25	25	25	25
9	*Zanthoxylum piperitum*	25, 50, 100, 200, 400	25	25	25	25	25	25
		Fig. 1	Fig. 2	Fig. 3	Fig. 4	Fig. 5	Fig. 6	Fig. 7

### Measurement of the antioxidant effect of essential oils in the absence of cells

An 2,2′‐azino‐bis‐(3‐ethylbenzothiazoline‐6‐sulfonate) (ABTS) assay was used to assess the free radical scavenging activities of the essential oils. To determine oil concentrations, working ABTS solution (Sigma‐Aldrich) was diluted to different extents with distilled water to an OD of 0.7. Working ABTS solution and essential oils were mixed in the ratio 99 : 1 and incubated for 10 min in the dark at 37 °C, and then, 200 μL of each mixture was transferred to a 96‐well plate. OD values were measured at 734 nm.

### Antioxidant effects of the essential oils against H_2_O_2_‐induced cell oxidative stress

Normal human dermal fibroblasts were seeded in 24‐well plates and cultured for 24 h in MEM containing 10% FBS and 1% streptomycin/penicillin at 37 °C. Cells treated with the essential oils at highest non‐toxic concentrations (refer to Table 2). To determine the antioxidant effects of the essential oils, 150 μm of hydrogen peroxide (H_2_O_2_; Sigma‐Aldrich), a negative control, was co‐treated with essential oils at a final concentration of 150 μm for 24 h. Cell viabilities were evaluated using the MTT assay.

### Effect of the essential oils on cell proliferation

A Cell Proliferation ELISA kit (cat. no. 11647229001; Roche Diagnostics, Indianapolis, IN, USA) 5‐bromo‐2‐deoxyuridine (BrdU) was used to measure the cell proliferation, as we previously described [27]. Skin fibroblasts were treated with the essential oils at various concentrations (Table 2). After 24 h, 100 μL of BrdU solution (100 μm) was added to each well for 4 h. Then, 1 mL of FixDenat was added to each well for 30 min to fix the cells. After fixation, the cells were incubated with BrdU antibody for 90 min at room temperature. After washing, 500 μL of substrate solution was added to cells for 20 min, and 125 μL of H_2_SO_4_ (1 m) was added. OD values were measured at 450 nm.

### Evaluation of the cell migration rate regulated by essential oils

Scratch migration assay was performed to analyze cell migration, as we previously described [27]. NHDFs were seeded into 24‐well plate and incubated for 24 h. The cells were scratched by a sterile 1 mL pipette tip and washed with PBS to remove detached cells. We added DMEM without FBS to the cells and treated the essential oils at the highest non‐toxic concentration (Table 2). The cells were incubated for 48 h and photographed at 0, 24, and 48 h. Scratched areas were visualized using a Nikon Eclipse Ts2 inverted microscope (Nikon Corporation, Tokyo, Japan).

### Measurement of the translational levels of ECM proteins regulated by essential oils

Protein samples of NHDFs treated with essential oils were extracted using Pro‐Prep solution (iNtRON Biotechnology, Seoul, Korea), according to the manufacturer's protocol. A bicinchoninic acid assay was used to determine protein concentrations. Proteins were then loaded and separated by 8% SDS/PAGE and transferred to nitrocellulose membranes using a wet transfer system. Membranes were blocked for 2 h with 5% skimmed milk in PBS containing 0.05% Tween‐20 (PBST) at room temperature. Subsequently, membranes were incubated with antibodies against collagen 1 (1 : 500; cat. no. sc‐293 182; Santa Cruz Biotechnology, Dellas, TX, USA), collagen 3 (1 : 500; cat. no. sc‐271 249; Santa Cruz Biotechnology), elastin (1 : 1000; cat. no. ab21736; Abcam, Cambridge, UK), and GAPDH (1 : 3000; cat. no. #2118; Cell Signaling Technology, Danvers, MA, USA), which served as the internal control, overnight at 4 °C. Blots were then incubated for 1 h with horseradish peroxidase‐conjugated secondary antibodies (1 : 5000; cat. nos. ADI‐SAB‐100 and ADI‐SAB‐300; Enzo Life Science, Farmingdale, NY, USA) in 5% skimmed milk in PBST for 1 h at room temperature. Luminol (Bio‐Rad, Hercules, CA, USA) was used to visualize antibody binding. Blots were scanned using Gel Doc 1000 version 1.5 (Bio‐Rad), and protein band intensities were normalized versus GAPDH.

### Contractile abilities of essential oils in three‐dimensional (3D) skin fibroblast cultures

A collagen gel contraction assay was used to assess contractile abilities of skin fibroblasts, as we previously described, to confirm the contractile activities of 3D cultured NHDFs (a skin model). In brief, a mixture of 4.7 mg·mL^−1^ collagen type 1, 10× PBS, and 1 N NaOH was prepared to obtain a collagen solution with a pH of 7.4 at a concentration of 4.13 mg·mL^−1^ [28]. Separately, NHDFs (2 × 10^5^ cells·mL^−1^) were prepared in DMEM without FBS. The collagen solution and NHDFs were then mixed to provide a final concentration of collagen of 1.5 mg·mL^−1^, and 300 μL/well of this gel mixture was dispensed into a 12‐well plate. The gels were then incubated for 1 h at 37 °C to allow collagen lattices to form. DMEM supplemented with 1% streptomycin/penicillin was added to the control and experimental groups, but DMEM supplemented with 5% FBS and 1% streptomycin/penicillin was used as positive control. The essential oils were then applied at the highest non‐toxic concentration (Table 2), and the lattices were physically detached from culture dishes. Gel images were captured at 0 and 24 h, and lattice areas were measured using image j software (National institutes of Health, Bethesda, MD, USA). The original areas of collagen gels were estimated immediately after treatment and used as internal controls. Contractile activities for each condition were determined in duplicate, and results were expressed as the mean ratio of changed diameter of collagen gel ± standard deviation (SD).

### Statistical analyses

Data are presented as the mean ± SD. Data were analyzed using one‐way analysis of variance (ANOVA; spss for Windows, 10.10, standard version; SPSS Inc., Chicago, IL, USA). Means obtained from three independent experiments were evaluated using one‐way ANOVA and Tukey's *post hoc t*‐test for multiple comparisons. A value of *P* < 0.05 was considered to indicate a statistically significant difference.

## Results

### IC_50_ values and highest non‐toxic concentrations of the nine essential oils for skin fibroblasts

**Fig. 1:**
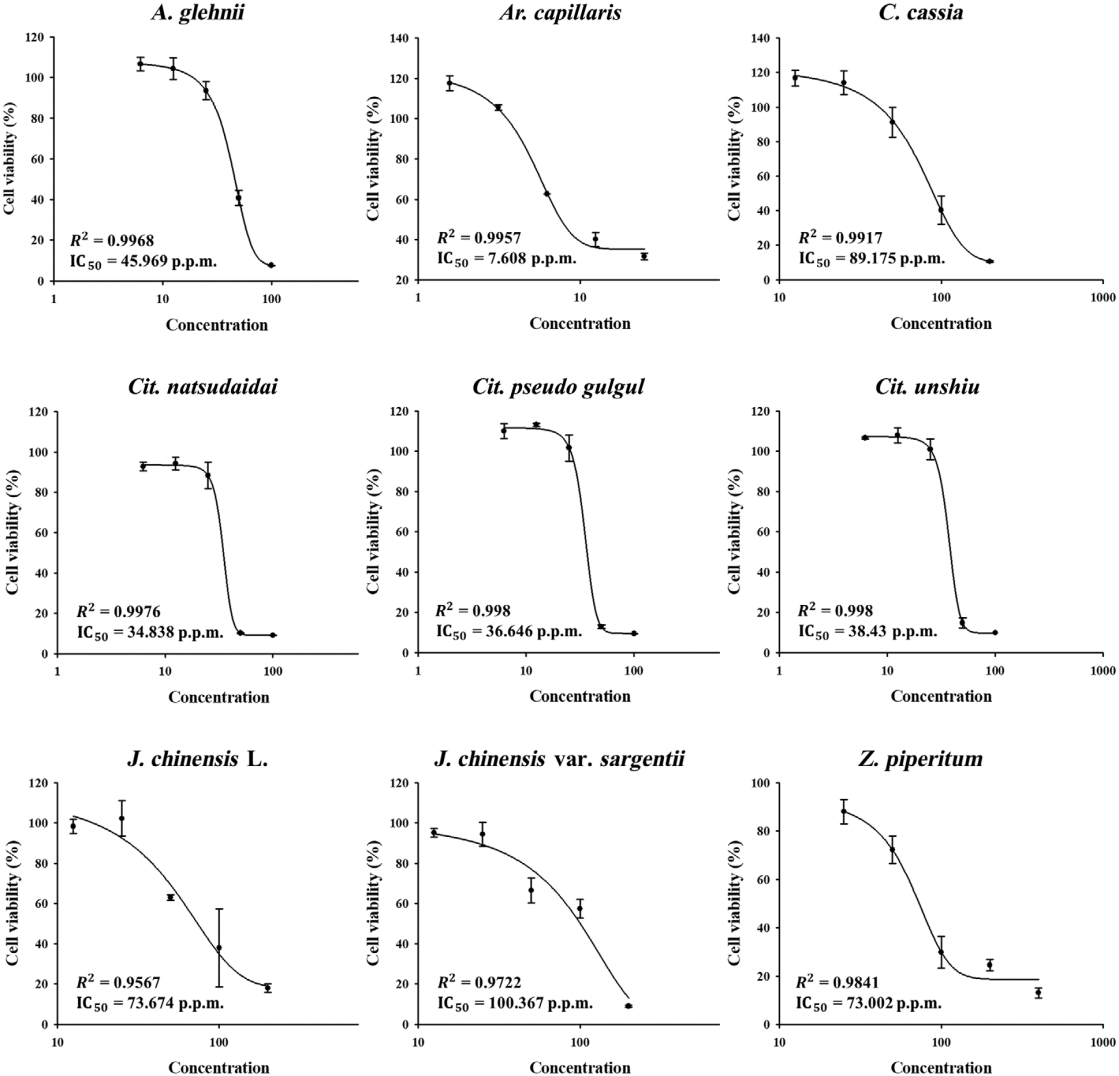
IC_50_ values of essential oils extracted from nine plants for skin dermal fibroblasts. Cells were treated with the nine essential oils in a wide range of concentrations, and cell viabilities were determined using the MTT assay (*n* = 3). Results are expressed as means ± SDs.

Normal human dermal fibroblasts were treated with essential oils extracted from *Aster glehnii* F. Schmidt E, *Artemisia capillaris*, *Zanthoxylum piperitum*, *Cinnamomum cassia*, *Juniperus chinensis* L., *Juniperus chinensis* var. *sargentii*, *Citrus natsudaidai* Hayata, *Citrus pseudo gulgul*, and *Citrus unshiu* in a wide range of concentrations (Table 2). We constructed cell viability curves and determined IC_50_ values using the MTT assay. The IC_50_ values for each essential oil were as follows: *J. chinensis* var. *sargentii*; 100.367 p.p.m. > *C. cassia*; 89.175 p.p.m. > *J. chinensis* L; 73.674 p.p.m. > *Z. piperitum*; 73.002 p.p.m. > *A. glehnii* F. Schmidt; 45.969 p.p.m. > *Cit. unshiu*; 38.43 > *Cit. pseudo gulgul*; 36.646 p.p.m. > *Cit. natsudaidai* Hayata; 34.838 p.p.m. > *Ar. capillaris*; 7.608 p.p.m. (Fig. 1). The test concentrations and highest non‐toxic concentrations of each essential oil were determined using these results and then used in various experiments as shown in Table 2.

### ABTS radical scavenging abilities of the essential oils

**Fig. 2:**
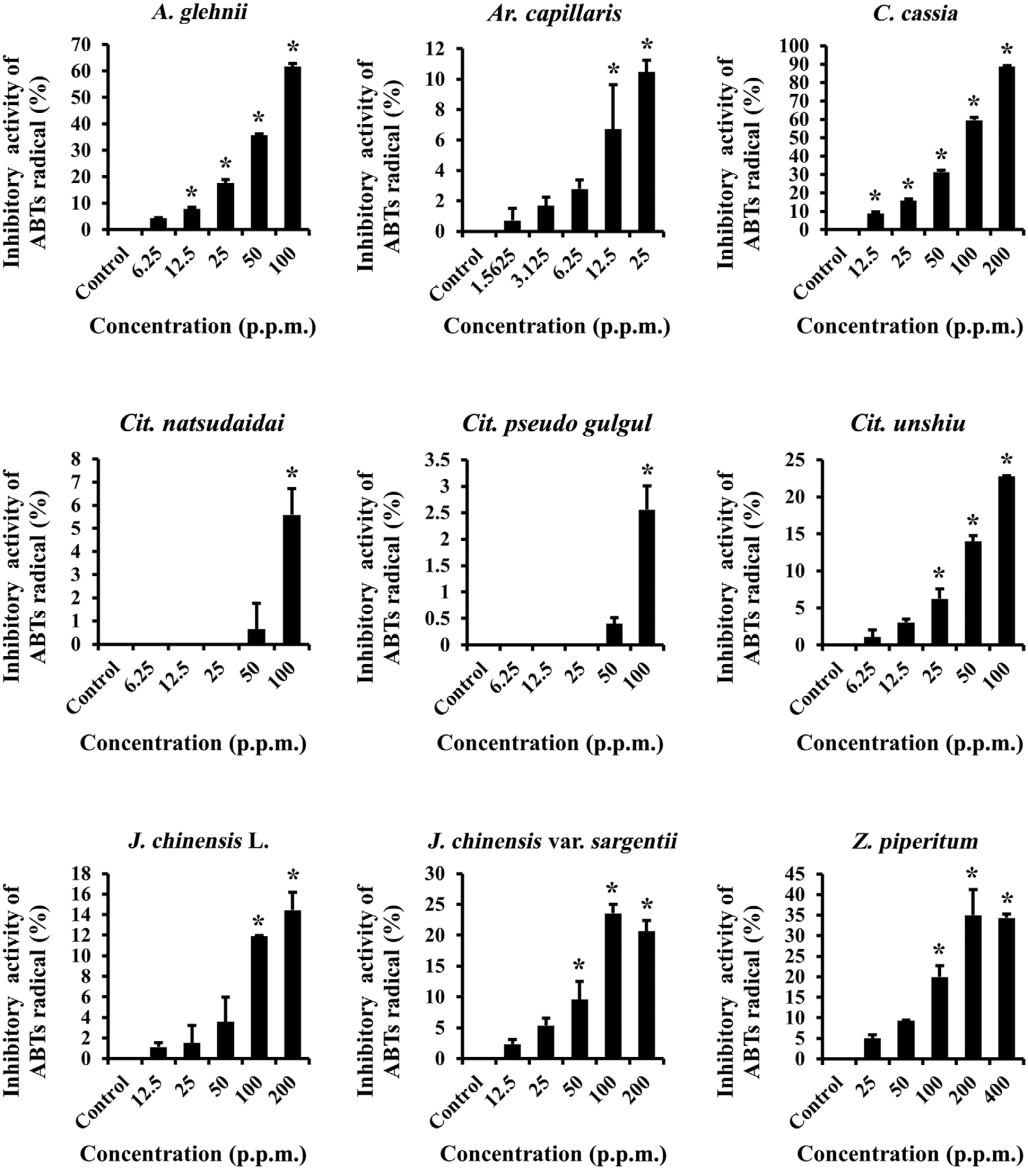
Antioxidant effects of essential oils in the absence of cells. The ABTS assay was used to assess the radical scavenging activity of the nine essential oils in a wide range of concentrations (*n* = 3). Results are expressed as means ± SDs. Statistical analysis: *t*‐test; *P < 0.05 significant difference compared to the control group.

We evaluated the antioxidant characteristics of the essential oils using the ABTS assay. Essential oils were used at the same concentration as those used for the MTT assay to validate that plant essential oils at the test concentrations established in the MTT assay exhibited antioxidant efficacy. Seven of the nine essential oils showed at least 10% radical scavenging activity at the highest concentrations; the essential oils of *Cit. natsudaidai* and *Cit. pseudo gulgul* were the two exceptions (Fig. 2). However, to assess more accurately the antioxidant effects of essential oils on cells, we performed MTT assay under H_2_O_2_‐induced oxidative stress in NHDFs.

### Protective effects of essential oils against H_2_O_2_‐induced oxidative stress

**Fig. 3:**
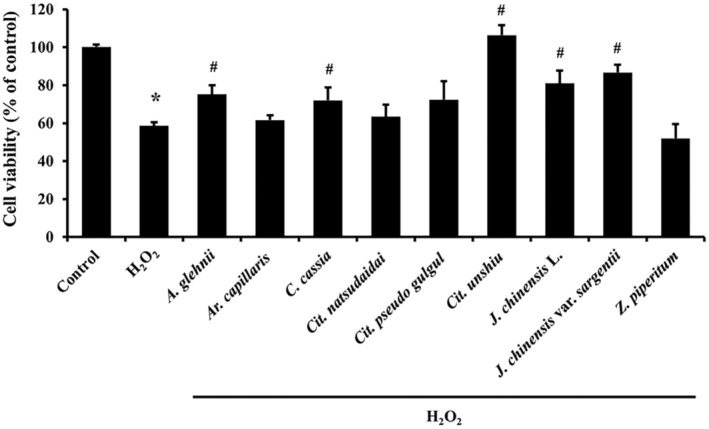
Antioxidant effects of essential oils on skin dermal fibroblasts. The protective effect of NHDFs on H_2_O_2_‐induced oxidative stress was investigated using the MTT assay (*n* = 3). Results are expressed as means ± SDs. Statistical analysis: *t*‐test; *P < 0.05 significantly different versus control group not treated with H_2_O_2_. ^#^ *P* < 0.05 significantly different with H_2_O_2_ treated group.

We used the MTT assay to examine the protective effects of essential oils against H_2_O_2_‐induced oxidative stress. NHDFs were treated with the nine essential oils at their highest non‐toxic concentrations (Table 2). One hundred and fifty micromolar H_2_O_2_ decreased skin fibroblast viability to 60% (Fig. 3). The essential oils extracted from *A. glehnii*, *C. cassia*, *Cit. unshiu*, *J. chinensis* L., and *J. chinensis* var. *sargentii* significantly recovered cell viability to 72–106%. However, essential oils extracted from *Ar. capillaris*, *Cit. natsudaidai*, *Cit. pseudo gulgul*, and *Z. piperitum* had no protective effect. Therefore, we further investigated the effects of these five essential oils.

### Effects of essential oils on NHDFs proliferation

**Fig. 4:**
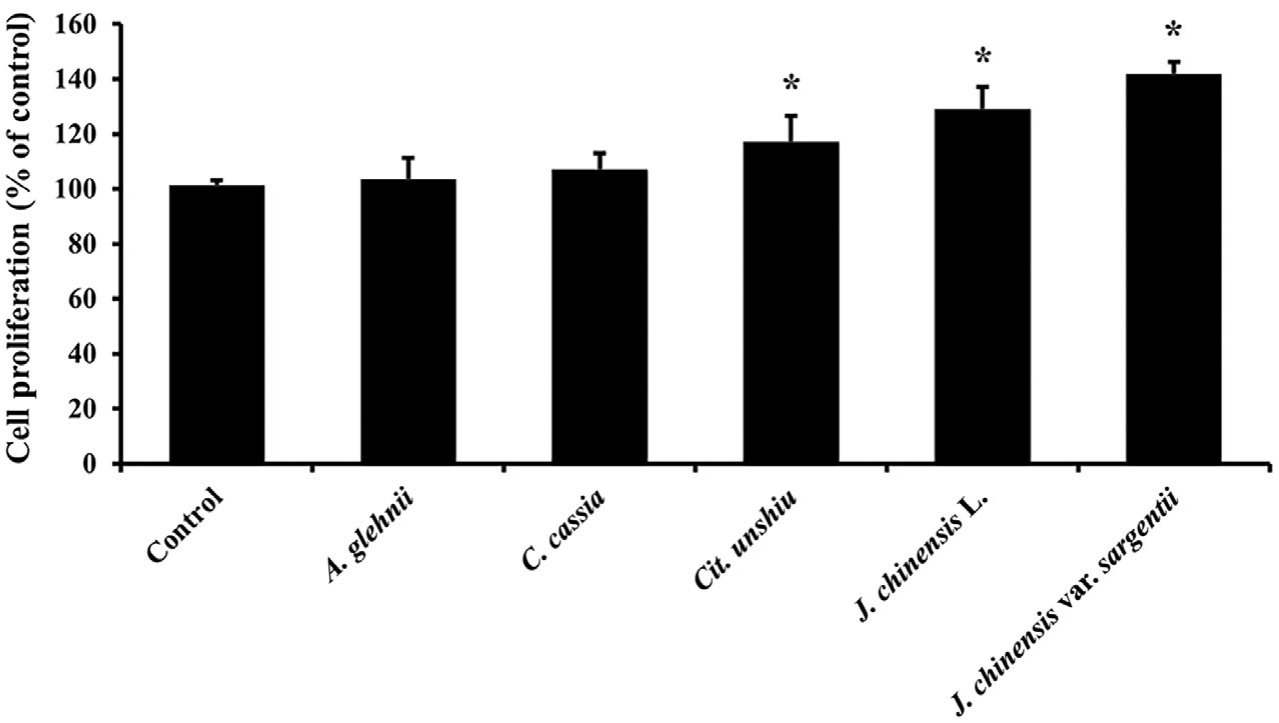
Effects of the five essential oils on skin fibroblast proliferation. The proliferations of NHDFs treated with the five essential oils were estimated using the BrdU assay (*n* = 3). Results are expressed as means ± SDs. Statistical analysis: *t*‐test; *P < 0.05 significant difference compared to the control.

The BrdU assay was used to estimate the proliferation of skin fibroblasts. NHDFs were treated with test concentrations of the essential oils of *A. glehnii*, *C. cassia*, *Cit. unshiu*, *J. chinensis* L., and *J. chinensis* var. *sargentii*. Essential oils extracted from *Cit. unshiu*, *J. chinensis* L., and *J. chinensis* var. *sargentii* significantly increased NHDFs proliferation versus controls (Fig. 4). The proliferation rate increased in the order *J. chinensis* var. *sargentii*, *J. chinensis* L., *Cit. unshiu*. However, *A. glehnii* and *C. cassia* did not regulate cell proliferation.

### Effects of the essential oils on migration of NHDFs

**Fig. 5:**
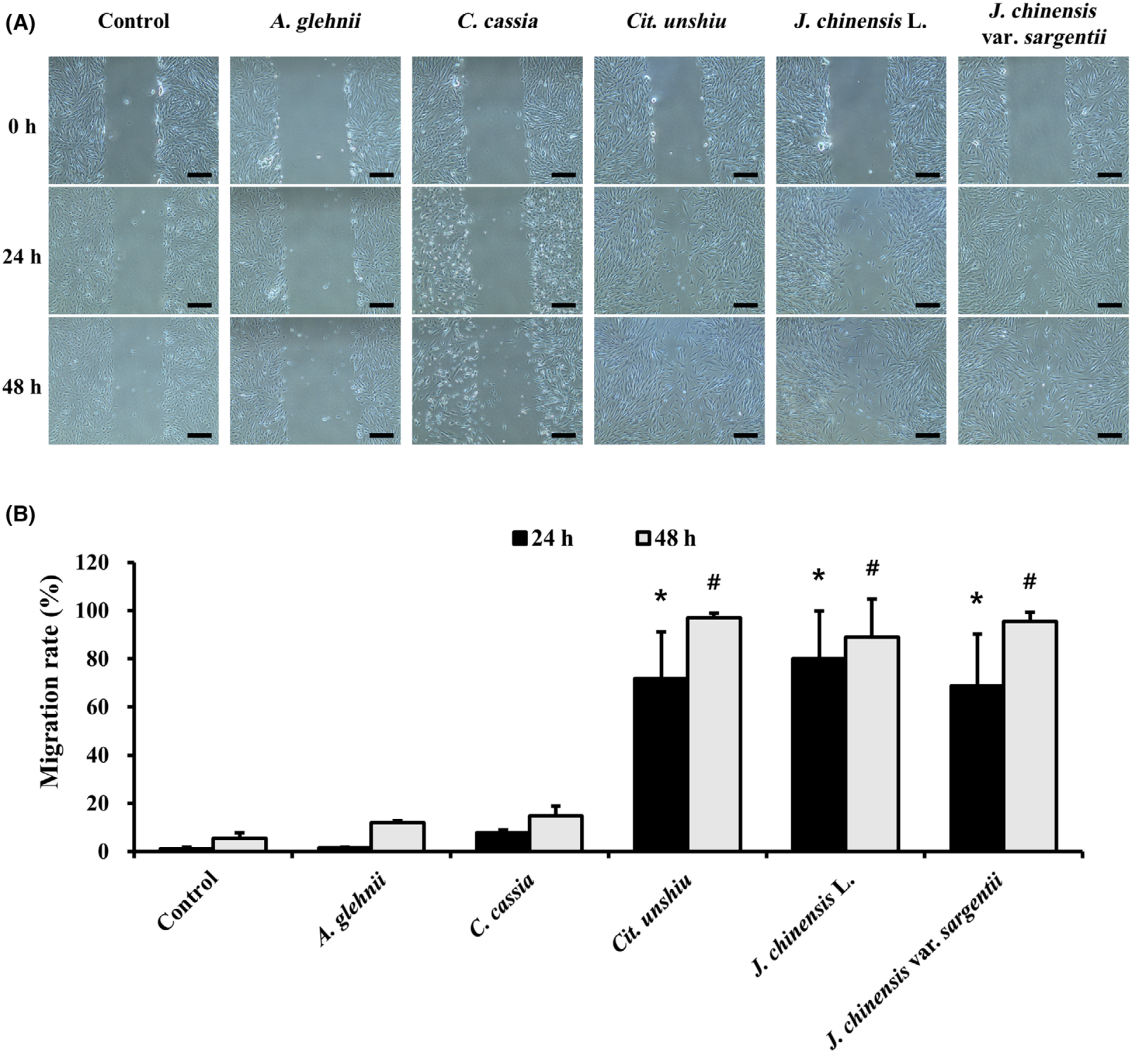
Cell migration of NHDFs treated with the five essential oils were analyzed using the scratch migration assay (A). Scale bars represent 200 μm. Migration rates are presented as graphs (B) (*n* = 3). Results are expressed as means ± SDs. Statistical analysis: *t*‐test; *P < 0.05 significant difference compared to the control (24 h). ^#^ *P* < 0.05 significant difference compared to the control (48 h).

A scratch migration assay was performed to determine whether the essential oils affect cell migration. The essential oils extracted from *A. glehnii* and *C. cassia* did not regulate migration rate compared to controls at both 24 and 48 h. However, the essential oils of *C. unchiu*, *J. chinensis* L. and *J. chinensis* var. *sargentii* increased migration rate significantly compared to controls at both 24 h (about 68–80%) and 48 h (about 88–96%) (Fig. 5A,B).

### Effects of the essential oils on ECM protein expression in skin fibroblasts

**Fig. 6:**
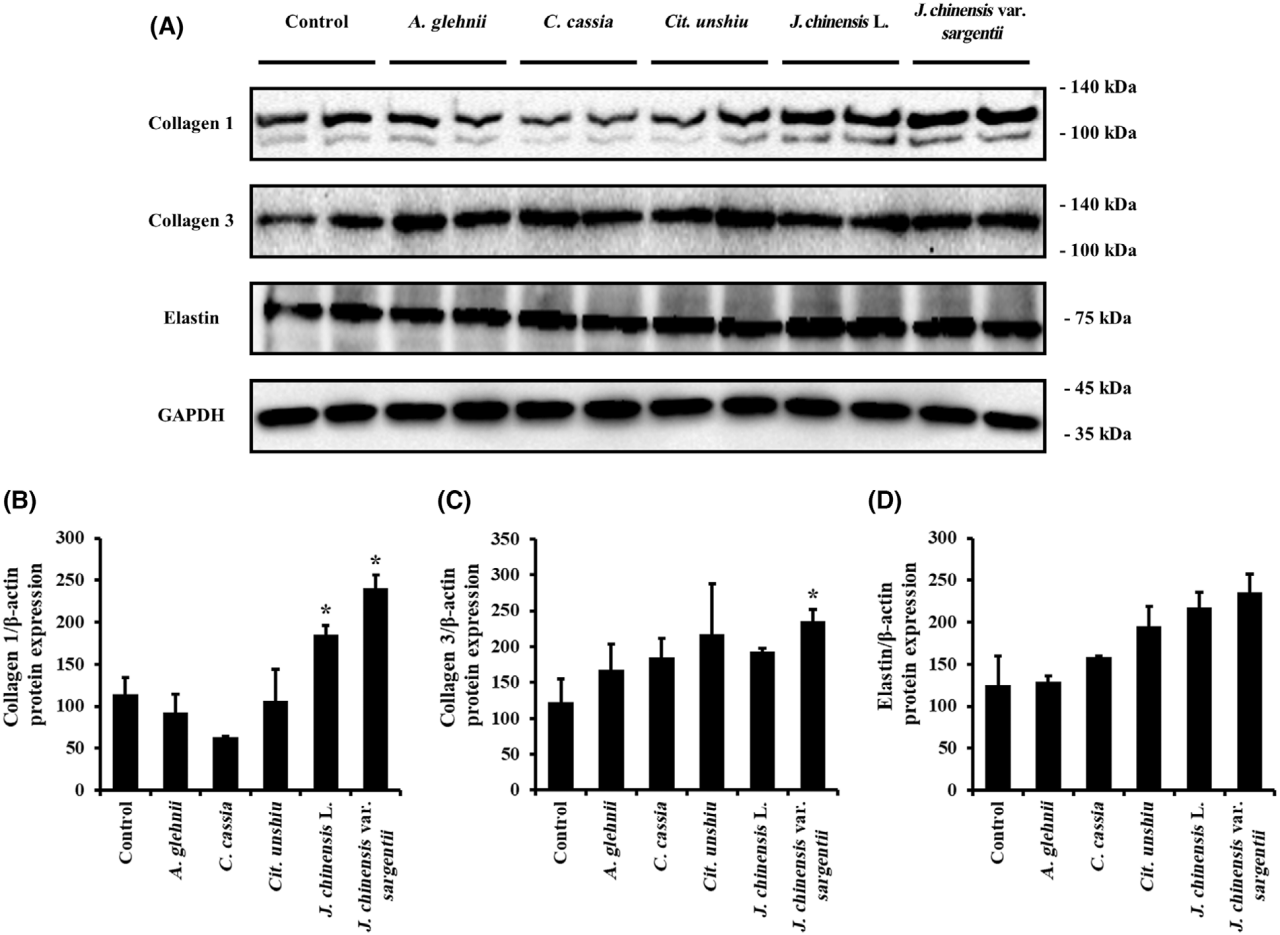
Translational levels of ECM proteins in NHDFs treated with the five essential oils. The protein expressions of ECM proteins were measured by western blot to evaluate protein production by fibroblasts treated with the five essential oils (A). The expression levels of (B) collagen 1, (C) collagen3, and (D) elastin are represented as graphs normalized versus GAPDH levels (*n* = 3). Results are expressed as means ± SDs. Statistical analysis: *t*‐test; *P < 0.05 significantly different versus controls.

Extracellular matrix protein levels were measured by western blot (Fig. 6A). The essential oils extracted from *J. chinensis* L. and *J. chinensis* var. *sargentii* significantly increased collagen 1 expression (Fig. 6B). The essential oils extracted from *A. glehnii*, *C. cassia*, *Cit. unshiu*, and *J. chinensis* L. increased expression of collagen 3 non‐significantly, but *J. chinensis* var. *sargentii* upregulated the expression of collagen 3 significantly (Fig. 6C). The expression of elastin was increased non‐significantly by essential oils extracted from *A. glehnii*, *C. cassia*, *Cit. unshiu*, *J. chinensis* L., and *J. chinensis* var. *sargentii* (Fig. 6D).

### Effects of the essential oils on 3D‐cultured skin fibroblasts

**Fig. 7:**
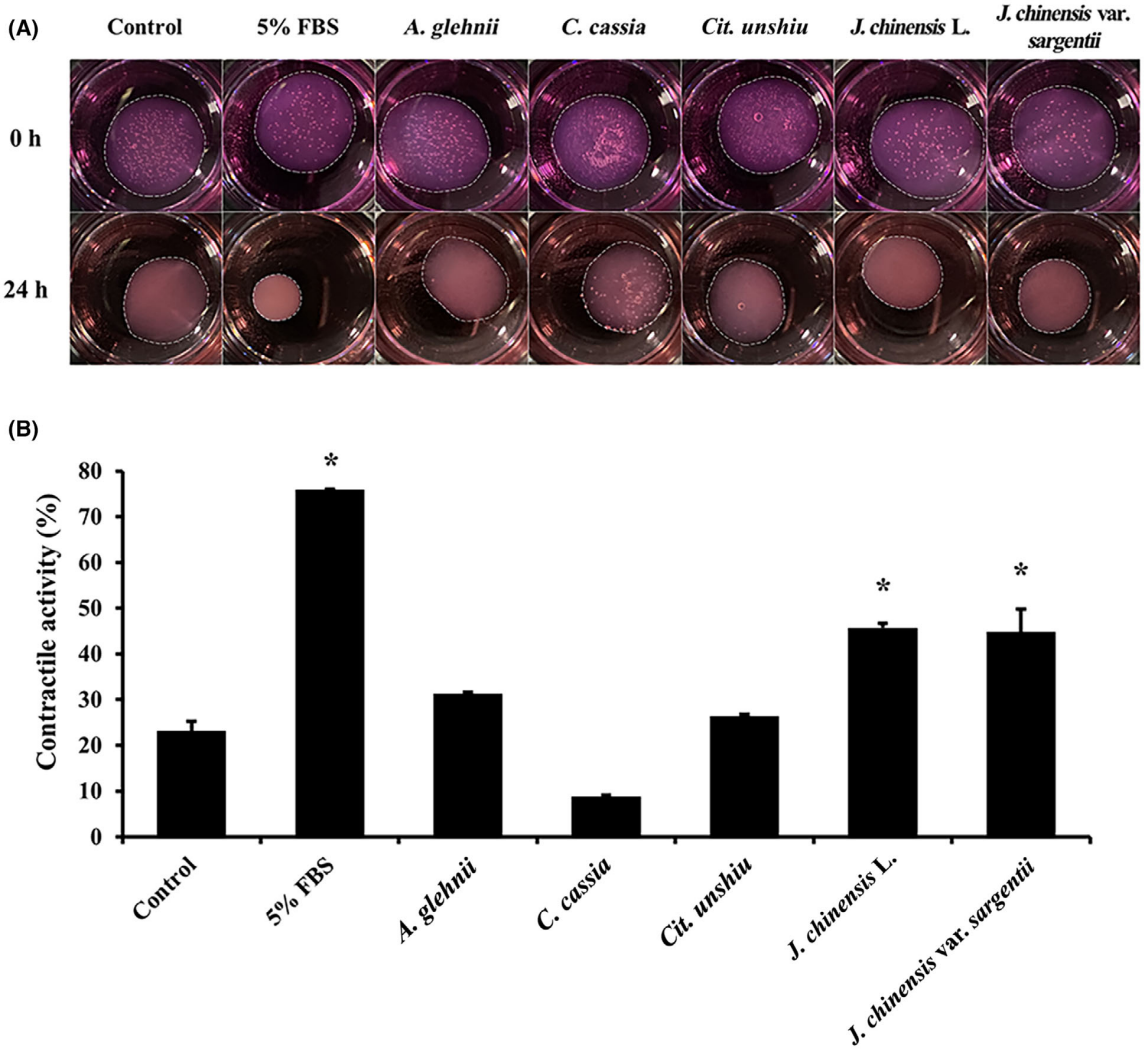
Gel contraction of 3D cultured NHDFs treated with essential oils (A). Contractile ratios are presented as histograms (B) (*n* = 3). Results are expressed as means ± SDs. Statistical analysis: *t*‐test; *P < 0.05 significant difference compared to the control.

Normal human dermal fibroblasts were cultured in a 3D environment using a collagen lattice to mimic the natural skin environment, and contractile abilities were assessed (Fig. 7A). The control group exhibited ~23% contraction of the collagen lattice, while the positive control group treated with 5% FBS showed a substantial contraction of ~76% (Fig. 7B). Essential oils extracted from *A. glehnii*, *C. cassia*, and *Cit. unshiu* did not induce significant shrinkage of the collagen lattice compared to controls. However, the essential oils of *J. chinensis* L. and *J. chinensis* var. *sargentii* induced a contraction of ~45%, indicating a significant increase in contractile activity versus controls.

## Discussion

Skin elasticity is an indicator of healthy skin and has a significant esthetic effect [29]. Skin elasticity is an emotionally important factor because unhealthy skin, which loses elasticity, can cause psychological distress [30]. Consequently, the medical and cosmetics industry continues to focus on developing healthier, environmentally friendly ingredients with minimal side effects [31, 32]. As a result, there is an increasing need to explore the possible utilization of medicinal plant resources and investigate their antioxidant properties and their impacts on extracellular matrix proteins associated with skin elasticity [33]. In this study, we evaluated the effects of essential oils extracted from nine medicinal plants on skin elasticity using skin fibroblasts.

Antioxidant activity is closely linked to the ability to remove ROS, which includes free radicals such as $O_{2}^{\cdot -}$ and OH^·^ and non‐radicals like H_2_O_2_ and ^1^O_2_ [34]. While ROS is necessary for normal cellular functions, excessive accumulation of ROS can lead to oxidative stress due to an imbalance between ROS production and antioxidant defense mechanisms. Oxidative stress can damage DNA, proteins, and lipids and contribute to the transformation of normal cells into cancer cells by causing mutations in key genes [35]. Furthermore, oxidative stress leads to the oxidation of DNA and mitochondria and subsequent cellular damage and cell death [36]. In our ABTS assay, we used a spectrophotometric method to assess antioxidant activity and found that seven of the nine plant essential oils exhibited at least 10% ABTS radical scavenging activity at the highest concentration tested [37]. Notably, *A. glehnii*, *C. cassia*, *Cit. unshiu*, and *J. chinensis* var. *sargentii* significantly scavenged radicals at concentrations below their IC_50_ values. In addition, the radical removal rate was increased in the order *C. cassia*, *A. glehnii*, *Cit. unshiu*, *J. chinensis* var. *sargentii*, and *J. chinensis* L. at the maximum non‐toxic concentration. Furthermore, *A. glehnii*, *C. cassia*, *Cit. unshiu*, *J. chinensis* L., and *J. chinensis* var. *sargentii* significantly inhibited the death of skin fibroblasts induced by H_2_O_2_, a representative ROS that induces oxidative stress leading to cell death [22, 36]. Therefore, these five plant essential oils demonstrated antioxidant properties irrespective of cellular activity.

Oxidative stress can impair cell viability, proliferation, and protein synthesis, and thus, intracellular antioxidant ability is beneficial as it acts to prevent cellular damage [38, 39]. *Cit. unshiu*, *J. chinensis* L. and *J. chinensis* var. *sargentii* significantly increased NHDFs proliferation and migration. The skin fibroblasts play a crucial role in maintaining skin elasticity by producing ECM proteins [40, 41]. When we examined ECM contents after treating NHDFs with essential oils, *J. chinensis* L. and *J. chinensis* var. *sargentii* were found to promote ECM protein production. In particular, collagen 1, collagen 3, and elastin were highly upregulated by the essential oils. Collagen is the most abundant ECM protein and plays an essential role in maintaining skin elasticity by preserving skin structure and enhancing physiological functions [42]. Also, elastin contributes to the unique elasticity of various connective tissues, plays a vital role in skin homeostasis, and is involved in dermal elasticity [43]. When NHDFs were cultured in a 3D environment, *J. chinensis* L. and *J. chinensis* var. *sargentii* significantly enhanced contractile activity [44]. Therefore, our findings show that *J. chinensis* L. and *J. chinensis* var. *sargentii* exhibit radical scavenging activity, act as antioxidants in NHDFs, and promote the survival, proliferation, and migration of these cells. Moreover, the activation of skin fibroblasts leads to the production of collagen and elastin, which are essential components of the ECM and increase the contractile activity of skin cells.

In this study, we did not confirm the compositional analysis of each plant essential oil, but we suggest that polyphenols or ascorbic acid may be the active ingredients in the oils. Polyphenols are a type of aromatic alcohol compounds found in plants. It has been reported that it prevents aging through its antioxidant effect, has the ability to protect DNA from damage due to exposure to active oxygen and has excellent functions to protect cellular proteins and enzymes, thereby lowering the risk of various diseases [45, 46]. Ascorbic acid is a water‐soluble vitamin found in plants, including citrus fruits. It is a powerful antioxidant that neutralizes free radicals and is reported to play an important role in collagen formation and is used as an ingredient in anti‐aging cosmetics [47].

Consequently, of the nine plant essential oils investigated in this study, those of *J. chinensis* L. and *J. chinensis* var. *sargentii* demonstrated medicinal potential as substances that enhance skin health and elasticity. These findings suggest that the essential oils of *J. chinensis* L. and *J. chinensis* var. *sargentii* could be used as novel ingredients that improve skin health and elasticity. Most commercially existing antioxidants are organic synthetic substances that could irritate the skin or have stability problems, while plant essential oils are mild and nature friendly. Therefore, these plant essential oils are expected to replace organic synthetic substances as natural antioxidants.

## Conflict of interest

The authors declare no conflict of interest.

## Author contributions

DSK, MJK, and B‐SA designed the experiments and wrote the manuscript. DSK, MJK, M‐JP, B‐JA, and S‐MA performed the experiments and analyzed the data. W‐JY and B‐SA confirmed the authenticity of all the raw data. M‐JP and B‐SA analyzed the data and revised the manuscript. All authors have read and approved the final manuscript.

## Data Availability

The datasets used and/or analyzed during the current study are available from the corresponding author on reasonable request.
